# On the Causes and Best Means of Preventing the Bowel Affections of Young Children

**Published:** 1882-07

**Authors:** N. S. Davis


### Article II.

### On the Causes, and best Means of Preventing the Bowel Affections of Young Children. By N. S. Davis, m.d., etc. Read to the Section on Diseases of Children at the Meeting of the American Medical Association, in St. Paul, June 8th, 1882; also, by request, to the Chicago Medical Society.

When it is remembered that one-third of the human race perish before they reach five years of age, and that a large percentage of these early deaths are the direct result of attacks of serous diarrhœa and cholera morbus, it will be conceded that no subject is more worthy of careful study than the pathology and prophylaxis of these affections. I mention these together because all measures designed to prevent disease must be intelligently adjusted either to the removal of the causes, or to a neutralization of their effects, or else they will fail to accomplish any useful purpose. Nearly all the public sanitary and hygienic measures of the present day are aimed at the removal or prevention of the causes of disease, both predisposing and exciting. But there are many influences which either predispose to or excite attacks of disease which are not under human control. The problem presented for consideration is not how to prevent or destroy them, but how best to shield the human system from their injurious effects. For instance, bad food may be destroyed and good substituted; bad air in dwellings may be changed by ventilation; soil, wet and filled with decomposable matter, may be drained and cultivated, but the meteorological conditions of the atmosphere, whether they relate to impurities, sudden and extreme changes or waves of continuous high or low temperature are not amenable to our control, and yet much can be done to mitigate or prevent their injurious effects. Nearly all the recent writers on the diseases of children class the cases of serous diarrhœa and cholera morbus in children under two years of age, usually called summer complaint and cholera infantum, with local inflammations under the general name of catarrhal gastro-enteritis, and while they all assert that these forms of disease are most prevalent and fatal during the warmest months of summer, they set forth as the chief causes, improper feeding, impure and changed milk, impure air, the process of dentition or teething, and overworked, badly fed and unhealthy mothers.

These causes are represented to produce gastric or intestinal indigestion, or both, which so increase the irritation of the mucous membranes as to cause a more or less rapid serous exudation into some part of the intestinal canal.

Indigestion is thus represented as the immediate cause of the catarrhal irritation, while the indigestion itself is attributed to impure and confined air, unwholesome food, cutting teeth, and the influence of badly fed, overworked, and unhealthy mothers and nurses. The great difficulty in the way of assenting to the correctness of this view of the etiology of the diseases under consideration, is, that all the alleged causes exist in nearly equal ratio at all seasons of the year, and in all large cities, without regard to their topographical surroundings. For instance, there is generally no more attention paid to ventilation and pure air in the winter than in the summer; there are certainly on the average no larger number of infants cutting teeth in July and August than in December and January; the milk distributed in all our cities is quite as liable to be diluted and adulterated in the cold as in the warm season of the year. But if we turn to the mortuary statistics of all the Northern and Eastern cities we shall find the prevalence of serous diarrhœa or summer complaint and cholera morbus in young children, limited almost wholly to three months of each year. For instance, in this city (Chicago) in the reports of the Health Department for 1877, we find 8 deaths recorded from cholera infantum in April, 6 in May, 23 in June, 246 in July, 163 in August, 69 in September,

13 in October, and only 2 in all the remaining five months of the year. Substantially the same ratio for the different months will be found in the records of every year. Again, the diseases under consideration are not only limited to a certain part of each year, but they are also limited mostly to cities and densely populated districts so located that there is a wide range of temperature between winter and summer, as well as protracted periods of continuous high temperature during the latter season. In Boston, New York, and Chicago the range of the mercury from the coldest days of winter to the warmest of summer is generally equal to 38° C. (100.5° F.), while in New Orleans and San Francisco it is less than half that number of degrees, and the nights are almost always cool. And from a comparison of the mortality statistics of all these cities, we find only *five* deaths from cholera infantum for every 10,000 of the inhabitants in San Francisco, and seven in New Orleans, while in Boston, New York and Chicago the ratio varies from twenty-five to thirty deaths for every ten thousand of their inhabitants. I have found no evidence that the milk distributed in San Francisco and New Orleans was better than that furnished in Boston and Chicago; neither is there any proof that the nurses and nursing mothers of the two first named cities are more free from mental and physical infirmities than those of the two last. And yet the ratio of deaths from cholera infantum is five times greater in the latter than in the former.

There must, therefore, be some efficient cause or causes for the prevalence of the disease not common to all large cities, nor active at all seasons of the year. For twenty-five years I have noted carefully, and part of the time made written memoranda, concerning the prevalence of the serous diarrhœa and cholera morbus in connection with the coincident meteorological conditions of the atmosphere.

For three years parallel records were kept by active general practitioners in Cairo, Davenport and Omaha. The detailed results of these observations and records were communicated to the Section on Practice of Medicine, etc., and may be found in the volumes of Transactions for 1875–77–79. They are sufficient to establish the following propositions:

First. That the prevalance of the affections under consideration is limited principally to July, August and September, commencing with the first wave of high atmospheric heat that continues days and nights for more than five days, which in the latitude of Chicago is sometimes the last week of June, but more frequently the first week in July, and continues more or less during the succeeding ninety days.

Second. That while the deaths from these affections in any city or given community will be nearly the same in the two first months after they begin in July and August, the date of the initial symptoms or beginning of the disease in three-fourths of all the cases will be in July, very few originating after the first of August. Many cases commencing in July continue until the months of August or September, causing wasting and death.

Third. That it is not simply high or extreme heat of temporary duration, such as that of a single day or any number of days, with cool nights, which favors the development of the disease, but continuous high temperature day and night for several days; and if, in addition to the heat, the air be stagnant from lack of winds or obstructions, as in large cities, or from defective ventilation, the effect is greatly increased. This explains why these affections are more numerous and fatal in cities than in rural districts, and why they prevail so little in even large cities located in warm climates provided the location be such as to afford cool breezes at night.

Fourth. That while the great majority of attacks which occur in any given summer are found to have their beginning in July, or during the first thirty or forty days after the first wave of protracted high temperature for the season, they are not equally distributed over the whole of the month. Having thus traced the origin of that part of infantile mortality caused by this disease, let us inquire for a moment how this combination of circumstances can affect the living human body. We have the physical law that the higher the temperature of the air the rarer it becomes, and the less oxygen is contained in a cubic inch. A person breathing a given quantity of air at a high temperature would receive less oxygen than at a lower temperature. Stagnant air becomes more rapidly exhausted than moving, and the physical law of expansion by increase of heat applies to the living as well as to dead matter, consequently high heat acting on the living body tends to increase the distance of the atoms from each other, and thereby lessens the force of vital affinity, while it increases the excitability or susceptibility to impression. The capacity of the blood for taking up oxygen or holding it in suspension depends much upon the proportion of saline elements it contains, and under a continuous high temperature the increase of cutaneous exhalation rapidly diminishes the free salts of the blood and lessens the capacity to receive the oxygen from the air cells of the lungs in exchange for its carbonic acid gas. Colitis and recto-colitis, or dysentery seldom occur until late in the season, when warm days are followed by cool nights and frequent changes from wet to cold occur; and even the indigestion which has been so generally suggested as a cause of summer complaint is itself the result of the impairment of natural gastric and intestinal secretions, and the increase of mere serous exudation—the primary fault not being so much in the quality of food as in the morbidly sensitive and relaxed condition of the whole inner surface of the digestive canal. The children are affected more than older persons because of the less mature development and greater sensitiveness of their gastric and intestinal mucous membranes and glandular structures, and their much more constant confinement indoors.

If this is correct, it indicates clearly that our efforts to lessen infant mortality from these diseases must embrace such measures as will secure for young children a better supply of fresh, pure air, for increasing the oxygenation and discarbonization of the blood and maintaining the activity of the vaso-motor nervous system; and as will counteract the effects of high temperature by increasing the general tonicity and lessening the excitability of the tissues generally. Measures for the first object must consist in securing better ventilation of dwellings, and especially nurseries and sleeping rooms during the warmest part of the summer, the sending of young children with their mothers and nurses from densely populated districts to moderately elevated healthy locations, or to floating hospitals, or receiving ships on large bodies of water during the special period of high heat. For accomplishing the second purpose, I know of no measures that are so efficient and at the same time within the reach of the poorest part of the population, as the judicious use of the sponge bath. Whenever the human system is relaxed and rendered morbidly sensitive by continuous high heat, causing the infant to be languid, restless and sometimes pale, a free bathing or sponging of the whole surface with water simply as cool as is comfortable, always produces a refreshing and invigorating influence, which continues from six to twelve hours. Consequently, if mothers and nurses could be so instructed by their family physician that during every wave or period of high atmospheric temperature in which the mercury did not fall below 70° during the nights, each child under two years of age should be regularly given a full sponge bath in the evening as well as in the morning, and their sleeping rooms as well ventilated as possible; such a course would diminish the attacks of serous diarrhœa and cholera infantum one-half, and consequently very greatly lessen the infant mortality from these affections. In the most oppressive evenings the wet bandage may be left around the trunk of the body after the bath.

It is well-known to every careful observer that a large majority of all the attacks of this form of disease show their first beginning during the last half of the night or early in the morning, owing to the long continuance of the high temperature, coupled with the more still and confined air of the night. The increased tone of the whole vascular system produced by the stimulant and tonic effect of a comfortably cool sponge bath on the function of the vaso-motor nerves, applied in the evening, would enable thousands of these little, restless sufferers to pass the whole night unharmed, when without it the dreaded sickness would begin. The views I have presented in regard to the causes and nature of the affections called summer complaint and cholera infantum also afford clear indications for the most rational and successful application of remedial agents in the treatment of those affections in all their grades of activity.

